# The Development of Vaginal Microbicides for the Prevention of HIV Transmission

**DOI:** 10.1371/journal.pmed.0020142

**Published:** 2005-05-31

**Authors:** Jonathan Weber, Kamal Desai, Janet Darbyshire, on behalf of the Microbicides Development Programme

## Abstract

Given that we still do not have an effective vaccine against HIV, the development of novel biomedical methods for preventing HIV transmission remains a top priority in controlling the HIV pandemic.

Microbicides are chemical agents used topically by women within the vagina in order to prevent infection by HIV and potentially by other enveloped viruses and sexually transmitted pathogens. Prototype microbicides are designed to be inserted prior to each act of sexual intercourse and could also be contraceptive, although most current potential microbicides are not. Several proof-of-principle phase III trials of candidate microbicides are currently in progress or are shortly to commence, and a definitive answer to their efficacy and safety is anticipated by 2008.

## Why Develop Chemical Barriers against HIV?

It is the unequivocal experience of the 20th century that the control of viral infections globally can best be achieved through the wide application of preventative vaccines. As discussed recently in *PLoS Medicine*, there is to be increased coordination and investment in a “Global Enterprise” to create an AIDS vaccine [1]. Disappointingly, however, in spite of much effort and investment, there is currently no proven vaccine against HIV, and no genuinely plausible candidate on the horizon. Richard Horton, editor of *The Lancet*, commented recently in the *New York Review of Books* that “one would be wise to plan the control of HIV infection globally over the next decade without assuming that a vaccine will be available” [2].

In the absence of a vaccine, novel biomedical methods for the prevention of HIV transmission assume far greater priority. Physical barriers such as the condom have been repeatedly demonstrated to have a high efficacy in the prevention of HIV transmission during sexual intercourse [3]. However, condoms remain almost exclusively under the control of the male partner, and in many societies women simply cannot negotiate condom use. A female-controlled method to prevent or reduce HIV risk is highly desirable, especially as this would allow sex education to promote mechanisms for both males and females, with the potential for an additive effect on the reduction of HIV transmission if used with a condom or other barrier methods. To be successful in reducing HIV transmission through being widely accepted and available, microbicides will need to be proven effective in phase III trials, and will need to be non-toxic and well tolerated, odour-less and colour-less, easily administered, and cheap to manufacture. [Fig pmed-0020142-g001]

**Figure pmed-0020142-g001:**
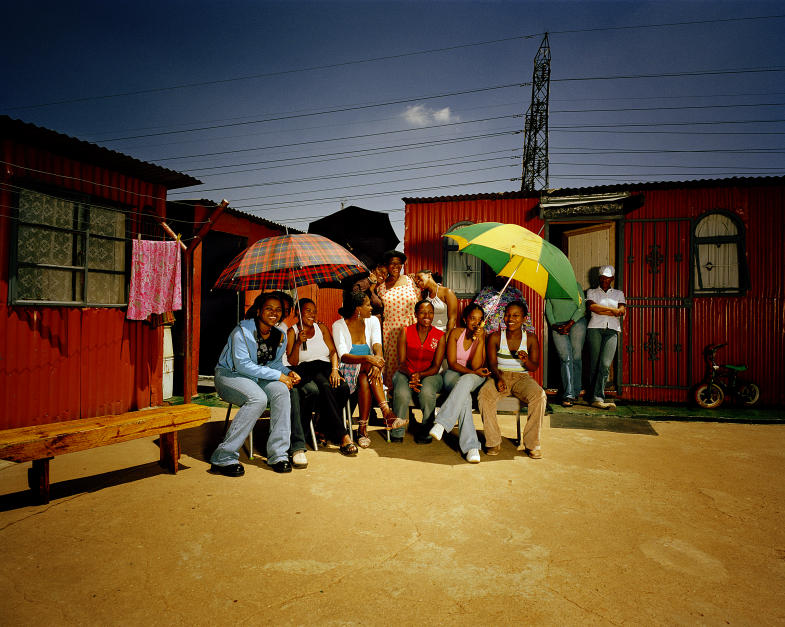
Women at a Microbicides Development Programme phase III trial site (Photo: Frank Herholdt; Copyright: © 2005 Microbicides Development Programme. This is an open-access photo distributed under the terms of the Creative Commons Attribution License, which permits unrestricted use, distribution, and reproduction in any medium, provided the original work is properly cited.)

## First-Generation Microbicides: Surfactants

The first vaginal microbicide to be studied was nonoxynol-9 (N9), an anionic surfactant initially developed in the 1960s as a contraceptive spermicide with lubricant properties, and latterly used extensively to coat latex condoms. N9 destroys the integrity of lipid bilayer membranes, and so has a virucidal action through disrupting the viral envelope.

The antiviral activity of N9 in vitro was first recognised in 1985 [4]. Evidence of activity in vivo was reported by Miller et al. in 1992 [5], with local N9 administration leading to reduction in transmission of simian immunodeficiency virus in a vaginal challenge macaque model. N9 has an IC50 of 2–5 ug/ml for both primary and laboratory-adapted HIV-1 strains (IC50 is the quantity of a substance that reduces HIV infection of cells in culture by 50%), and it is active against diverse HIV genotypes in vitro. However, the antiviral effect of N9 is non-specific, with the CC50 (the quantity of a substance required to damage cells in culture such that active uptake of tritiated thymidine or other biomarkers is reduced by 50%) and IC50 occurring at similar concentrations.

Early experience in placebo-controlled field studies by Joan Kreiss et al. in 1992 in Kenya [6] and by Ron Roddy et al. in 1998 among commercial sex workers in Cameroon [7] suggested that N9 may be associated with local vaginal toxicity, including ulcerations, without clear evidence of efficacy against HIV transmission. A phase I safety study of N9 in healthy women volunteers in London showed that regular administration of N9 led to histological evidence of vaginal inflammation, including increased CD4^+^ T lymphocytes and macrophages [8]. The final phase III multi-centre randomised placebo-controlled trial (COL 1492) of N9, undertaken by the United Nations Joint Programme on HIV/AIDS in 2002, showed that N9 had no efficacy in preventing HIV transmission [9]. Indeed, the transmission rate was marginally higher in the N9 group, and it was considered that this might be related to the local vaginal toxicity seen in earlier studies.

**Figure 1:**
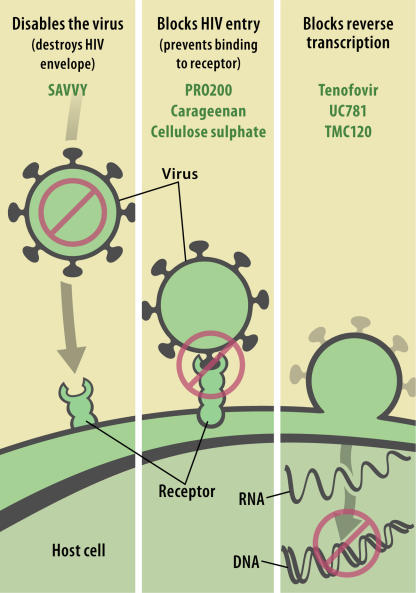
Sites of Action of Candidate Microbicides (Illustration: Giovanni Maki)

More recently, the company Biosyn has developed a new surfactant microbicide, SAVVY (Figure 1), which is thought to be significantly less cytotoxic than N9 while retaining antiviral activity, and which is currently in phase III trials in sites in West Africa. SAVVY is believed to exert its virucidal activity through disruption of the HIV envelope lipid bilayer via the same mechanism as N9, although the basis of its lower cytotoxicity is not reported [10].

## Second-Generation Microbicides: Blocking HIV Binding

The problems with N9 strongly suggested that for new vaginal microbicides to be effective, these agents would need to be largely devoid of local vaginal toxicity. The first class of agents to be systematically explored (in 1992) were agents that blocked the binding of HIV-1 to target cells in vitro [11]. The anti-HIV activity of polyanions had first been described in 1987 [12], but these agents had too high a molecular weight to be orally absorbed, and interfered with clotting if given parenterally, and hence they were not developed for antiviral therapy.

Candidate agents in this class (i.e., agents that block HIV binding) now include several high molecular weight anionically charged sulphated polymers such as PRO 2000 (a naphthalene sulphonate polymer), carageenan (a naturally occurring sulphated sugar polymer), and cellulose sulphate (Figure 1). These three agents have potent anti-HIV activity in vitro against the two major classes of HIV-1 isolates using both the CCR5 and the CXCR4 co-receptors. Unlike N9, these agents have a very low toxicity, and the therapeutic index (ratio of CC50 to IC50) is above 10,000.

PRO 2000 has been extensively studied in vivo in the simian-human immunodeficiency virus macaque vaginal challenge model, with 50%–90% protection against infection depending on experimental conditions [13]. The absence of absorption of these high molecular weight agents is a positive benefit for intra-vaginal use, as very high local concentrations of these polyanions have been shown to be tolerable in animal models and in human phase II trials [14]. PRO 2000, carageenan, and cellulose sulphate are all in, or will shortly be in, phase III trials in women at risk of HIV infection in Africa (see Table 1).

**Table 1:**
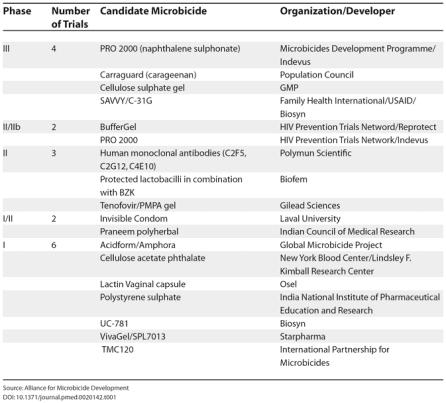
Status of Clinical Trials of Current Microbicide Compounds

Source: Alliance for Microbicide Development

The healthy vagina is maintained at low pH and microbially populated with hydrogen-peroxide-producing lactobacilli. There is some evidence that maintenance of this healthy vaginal milieu may protect against infection by HIV and other sexually transmitted infections; all current microbicides are buffered to pH 4.5 in order to promote vaginal health, and all have been investigated for their ability to sustain vaginal lactobacilli in vivo [15]. One candidate microbicide, “BufferGel”, whose mechanism of action is specifically based on the maintenance of a low vaginal pH, is being assessed in parallel with PRO 2000 in a phase IIb trial ([16]; see Table 1).

## Third-Generation Microbicides: Topical Antiretrovirals

The polyanions meet many of the desirable properties of an ideal vaginal microbicide, being cheap to manufacture, active in vitro and in macaque vaginal challenge experiments, and safe and tolerable in vivo in phase II trials. However, they rely on charge for their effect, and they are non-specific for enveloped viruses and may all be slightly less active against CCR5-using primary virus isolates, the major transmitted variant of HIV-1. Ongoing phase III trials will ultimately determine whether their activity and tolerability translate into efficacy. It is too premature to assume success with the second-generation agents, and HIV-specific microbicide agents are currently being developed in phase I and II clinical trials.

Tenofovir, a nucleotide analogue reverse transcriptase inhibitor antiretroviral drug with a long half-life, has been successfully introduced as an oral agent for HIV infection. Studies of tenofovir in macaques suggest that pre-dosing may prevent simian immunodeficiency virus infection, and a gel formulation has been developed for vaginal use. A multi-centre phase II trial of vaginal tenofovir gel is planned for 2005.

Non-nucleoside reverse transcriptase inhibitors (NNRTI) represent a range of chemical structures with direct binding to the active site of HIV-1, and efavirenz and nevirapine are very successful NNRTIs used in combination antiretroviral therapy. Some identified NNRTIs are highly lipophilic, unsuitable for oral administration but potentially useful for intra-vaginal use as microbicides. Of these, UC781 and TMC120 are being formulated and developed as vaginal gels (Figure 1), with the aim of entering phase III trials in 2006 or 2007.

Finally, data have recently been reported on the prevention of simian-human immunodeficiency virus transmission in the macaque vaginal challenge model by the use of monoclonal antibodies to HIV-1 [17] and by a topical small-molecule CCR5 inhibitor, PSC-RANTES [18]. In both cases, the quantity of the agent required to prevent transmission in vivo was considerably greater than that required in vitro. The use of these highly specific agents in combination with other second- or third-generation microbicides may address this issue. However, monoclonal antibodies and peptides alone are never likely to meet the requirement for a microbicide to be cheaply manufactured.

## Phase III Trials of Vaginal Microbicides

Clinical trials of the efficacy of microbicides present particular problems over and above those encountered for trials of preventative HIV vaccines. Trial populations must be identified with an HIV incidence of at least 2% if sample size is to be feasible. However, this high level of HIV transmission must be vigorously addressed though counselling, screening for and treatment of sexually transmitted infections, and provision of condoms and health education. Adherence to use of the microbicide will be central to a successful outcome, and these agents will need to be used to protect every risky act of intercourse. As with male condoms, adherence is unlikely to be complete, and may reduce over time. Thus, trials designed to assess proof of efficacy should be for as short an intervention period as possible in order not to dilute any antiviral effect of the microbicide through the likely reduction in adherence over time.

Further, non-vaginal exposure to HIV such as through frequent anal intercourse or the use of non-sterile needles, will reduce the effect of a microbicide. Thus, considerable behavioural data will need to be collected at these phase III trials for the results to be fully interpreted and understood. These trials will therefore be large, requiring multiple sites in high-incidence regions, and they will be long in duration and hence expensive.

Phase III trials of novel microbicide products undertaken in resource-poor regions in large numbers of women are financially challenging, inevitably costing upwards of $US50 million depending on sample size. In the absence of commercial investment, this burden falls on the charitable and/or public sector. It is highly likely that because of the high costs, phase III trials will be the major rate-limiting step to new product development and testing.

In the long term, the effectiveness of microbicides will be highly dependent on continued high-level adherence, and this will need to examined in longer term trials As the safety and tolerability of these new materials will continue to be paramount, some participants even in shorter trials will need longer follow-up for safety. Ultimately, it is possible that adherence could be enhanced through novel depot methods for the sustained delivery of microbicides within the vagina without the need for dosing prior to each act of intercourse. For example, the development of resident vaginal rings allowing sustained slow release of the active agent over weeks or months would overcome the key issue of adherence.

## Advocacy, Funding, and Public–Private Partnerships

To date, there has been little interest from the pharmaceutical industry, outside of biotech companies, in the development of vaginal microbicides, and the burden of funding has fallen on the public and charitable sectors. This reliance on non-private funding has led to the new agents being developed in the academic sector, which has probably slowed progress even though the approach has been highly cost effective. However, microbicide research has attracted considerable political attention because of the urgency of the HIV epidemic, the plight of vulnerable women in high-incidence regions, and the delays in progress towards an HIV vaccine. A product-development public–private partnership has been established, the International Partnership for Microbicides, which is establishing clinical trial sites and is developing TMC120. Other advocacy groups include the Global Campaign for Microbicides and the Alliance for Microbicides, which ensure that pressure is maintained for funding the development of an effective microbicide.

Although it is hoped that one or more of the second-generation potential microbicides will have a positive risk–benefit ratio, they may have only partial efficacy, perhaps being as low as 35% effective. Indeed, the effect of these second-generation agents may be even lower when they are used outside of a clinical trial. Alternative approaches that require less attention and action from users—such as vaginal rings, more potent, longer acting products, or combinations of agents that might need to be used only once a day—could increase the effectiveness of microbicides and therefore have a much greater effect on HIV transmission.

However, modelling suggests that even a microbicide shown to be only partially effective, if used by women in concert with the promotion of condoms for men, would have a beneficial effect on the reduction of HIV transmission at a population level. In a sub-Saharan setting where endemic HIV prevalence is currently 10.8%, the introduction of a microbicide of 50% efficacy covering 50% of sex acts in high-risk women could achieve a population-wide reduction in HIV prevalence to 8.1% after 20 years. Concurrent promotion of condoms additionally covering 50% of sex acts in high-risk men could potentially achieve a prevalence as low as 1.4% (see simulations; Table 2). In the continued absence of an effective HIV vaccine, a partially active microbicide would still be a highly desirable intervention.

**Table 2:**
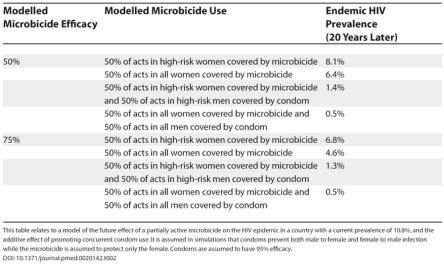
Modelling Microbicide Effectiveness

This table relates to a model of the future effect of a partially active microbicide on the HIV epidemic in a country with a current prevalence of 10.8%, and the additive effect of promoting concurrent condom use. It is assumed in simulations that condoms prevent both male to female and female to male infection while the microbicide is assumed to protect only the female. Condoms are assumed to have 95% efficacy.

## Abbreviations

N9: nonoxynol-9
NNRTI: non-nucleoside reverse transcriptase inhibitor
